# Implementation of a Miniaturized Planar Tri-Band Microstrip Patch Antenna for Wireless Sensors in Mobile Applications

**DOI:** 10.3390/s22020667

**Published:** 2022-01-16

**Authors:** Ahmed Saad Elkorany, Alyaa Nehru Mousa, Sarosh Ahmad, Demyana Adel Saleeb, Adnan Ghaffar, Mohammad Soruri, Mariana Dalarsson, Mohammad Alibakhshikenari, Ernesto Limiti

**Affiliations:** 1 Department of Electronics and Electrical Communication Engineering, Faculty of Electronic Engineering, Menoufia University, Menouf 32952, Egypt; elkoranyahmed@el-eng.menofia.edu.eg (A.S.E.); alyaanehru27@gmail.com (A.N.M.); 2 Department of Electrical Engineering and Technology, Government College University Faisalabad (GCUF), Faisalabad 38000, Pakistan; 3 Department of Signal Theory and Communications, Universidad Carlos III de Madrid, Leganés, 28911 Madrid, Spain; 4 Faculty of Engineering, Kafrelsheikh University, Kafrelsheikh 33516, Egypt; demyanasaleeb@eng.kfs.edu.eg; 5 Department of Electrical and Electronic Engineering, Auckland University of Technology, Auckland 1010, New Zealand; aghaffar@aut.ac.nz; 6 Technical Faculty of Ferdows, University of Birjand, Birjand 9717434765, Iran; mohamad.soruri@birjand.ac.ir; 7 School of Electrical Engineering and Computer Science, KTH Royal Institute of Technology, SE 100-44 Stockholm, Sweden; 8 Electronic Engineering Department, University of Rome “Tor Vergata”, Vial Del Politecnico 1, 00133 Rome, Italy; limiti@ing.uniroma2.it

**Keywords:** triband antenna, wireless sensors, planar patch antenna, mobile applications, DCS, WLAN, WiMAX

## Abstract

Antennas in wireless sensor networks (WSNs) are characterized by the enhanced capacity of the network, longer range of transmission, better spatial reuse, and lower interference. In this paper, we propose a planar patch antenna for mobile communication applications operating at 1.8, 3.5, and 5.4 GHz. A planar microstrip patch antenna (MPA) consists of two F-shaped resonators that enable operations at 1.8 and 3.5 GHz while operation at 5.4 GHz is achieved when the patch is truncated from the middle. The proposed planar patch is printed on a low-cost FR-4 substrate that is 1.6 mm in thickness. The equivalent circuit model is also designed to validate the reflection coefficient of the proposed antenna with the S_11_ obtained from the circuit model. It contains three RLC (resistor–inductor–capacitor) circuits for generating three frequency bands for the proposed antenna. Thereby, we obtained a good agreement between simulation and measurement results. The proposed antenna has an elliptically shaped radiation pattern at 1.8 and 3.5 GHz, while the broadside directional pattern is obtained at the 5.4 GHz frequency band. At 1.8, 3.5, and 5.4 GHz, the simulated peak realized gains of 2.34, 5.2, and 1.42 dB are obtained and compared to the experimental peak realized gains of 2.22, 5.18, and 1.38 dB at same frequencies. The results indicate that the proposed planar patch antenna can be utilized for mobile applications such as digital communication systems (DCS), worldwide interoperability for microwave access (WiMAX), and wireless local area networks (WLAN).

## 1. Introduction

The need for mobile communication systems has risen dramatically in the last decade, and it continues to rise. The important standards in mobile communication are GPS, Wi-MAX, and WLAN. These wireless applications require efficient small size antennas. Portable antenna technology has grown, along with cellular and mobile technologies [1,2,3,4]. 

The antenna is a crucial component of a communication system, and it is the device that transfers electromagnetic wave into free space in transmitting modes and vice versa [5,6,7]. Different antennas are required to support multiband systems. Multiband antennas play an important role in mobile communications, because they can be used in various frequency bands such as DCS, Wi-Fi, WLAN bands (802.11 b/n/g), and WiMAX (IEEE 802.16) [8,9,10]. There are different types of antennas such as PIFA, dipole, monopole, etc., that can be used in these applications. In general, the microstrip patch antenna (MPA) is an essential part of the communication system since it possesses several distinct and appealing characteristics. Compact size, low-cost, simple structure, minimal weight, ease of manufacture, and a wide bandwidth are some of these characteristics. MPA is a common choice for systems with a variety of features and the capacity to support many frequency bands at the same time [11]. The size of the patch antenna is determined by the dielectric constant of the substrate [12,13]. MPAs are chosen as the best antenna design because they are easy to implement with integrated circuits [14,15,16]. Such an antenna should provide high gain, wide impedance bandwidth, suitable return loss, and improved efficiency [17,18]. The MPA’s biggest drawback is its limited impedance bandwidth. There are numerous methods for resolving this issue [19,20]. The U-slots technique is a popular patch-etching technique for obtaining multiband operation, and it was primarily utilized for increasing bandwidth [21]. Several articles have discussed various design and analytic techniques for improving performance [22,23,24]. The E-shaped, H-shaped, and U-slotted patch MPAs are very common with interesting characteristics [25,26,27,28]. Many researchers have recently reported on the design of MPAs that include slots or many layers. Li et al. [25] reported double and triple resonant frequencies for single and double patch antennas with an air substrate. The performance of equal-sized rectangular patch antennas with and without two included L-shaped strips was compared by Khunead et al. [29]. Prasad et al. [30] reported a triband heart-shaped MPA for wireless communications that can cover the 2.4, 5.4, and 7.6 GHz frequency bands. Darimireddy et al. [21] reported that wide bandwidth and triple bands at 1.6 GHz, 1.9 GHz, and 3.8 GHz are achieved by using a combination of dual U-slot and multiple layers. Ghalibafan et al. [31] described a multiband microstrip patch antenna for WLAN, Wi-MAX, and X-band use. Chitra et al. [32] introduced a new design technique for a microstrip patch antenna with an E-slot and an a-slot at the radiator’s edge for Worldwide Interoperability for Microwave Access (WiMAX) application by using the Rogers Duroid 5880 substrate.

Osama et al. [26] reported a Double U-slot rectangular patch antenna for multiband applications to obtain thee resonant frequencies. Asif et al. [27] designed a printed microstrip patch antenna with two rectangular U-shaped parasitic elements to operate three resonant frequencies at 6.2, 4.52, 6.9 GHz. Gupta et al. [33] reported a multiband frequency with dimensions 60 × 55 × 1.59 mm^3^. The developed antenna efficiently operates at 4.3 GHz, 5.0 GHz, 6.1 GHz, 7.4 GHz, 8.9 GHz, and 9.2 GHz. Roopa et al. [34] proposed a square fractal antenna, which can operate in multiband frequency in the range of 2 GHz to 8.2 GHz with dimension 70 × 70 × 1.58 mm^3^. Dabas et al. [35] presented a microstrip patch antenna for wireless application, which can operate at 2.313, 2.396, and 2.478 GHz with dimension 70 × 70 × 1.6 mm^3^. Mazen et al. [36] reported a microstrip antenna to operate at multiple frequencies with dimensions 94 × 76 × 3.18 mm^3^. 

Wireless sensor networks (WSNs) allow innovative applications and involve non-conventional design models due to some limitations. Antennas in wireless sensor networks (WSNs) have several advantages, such as enhanced capacity of the network, longer range of transmission, better spatial reuse, and lower interference. The reliability requirements and energy concerns make that antenna technology more advantageous. The proposed antenna is tri-band and works at 1.8 GHz, 3.5 GHz, and 5.4 GHz frequency bands. At the frequency of 1.8 GHz, it is applicable for digital control systems (DCS); at 3.5 GHz, it is applicable for the worldwide interoperability for microwave access (WiMAX); and at 5.4 GHz, it is applicable for wireless local area network (WLAN). In this article, a planar MPA fed by a coaxial probe is proposed for the applications in mobile communications. In the suggested design, we used horizontal slots, a single patch, and a single layer to make fabrication simple and easy. The radiating planar patch is located at the center of the substrate backed by a partial ground plane. The planar patch consists of two F-shaped resonators with a truncated patch from its middle point to obtain multiband operation. The proposed antenna is implemented on an FR-4 substrate with the following characteristics: height = 1.6 mm, dielectric constant *ε_r_* = 4.3, and loss tangent of 0.025. In this study, a new approach for the planar patch antenna is utilized in order to obtain triband characteristics covering DCS (1.4–2 GHz), WiMAX (3.4–3.8 GHz), and WLAN (5.2–5.6 GHz) frequency bands. The size of the antenna is calculated as 60 mm × 50 mm. The proposed antenna is simulated using CST Microwave Studio (CST MWS) software. The outline of the paper is as follows: The design principles and recommended antenna geometry are described in Section 2. Section 3 explains the proposed measurement findings, while Section 4 presents conclusions. 

## 2. Antenna Design Methodology

### 2.1. Proposed Single Antenna Design

**Figure 1:**
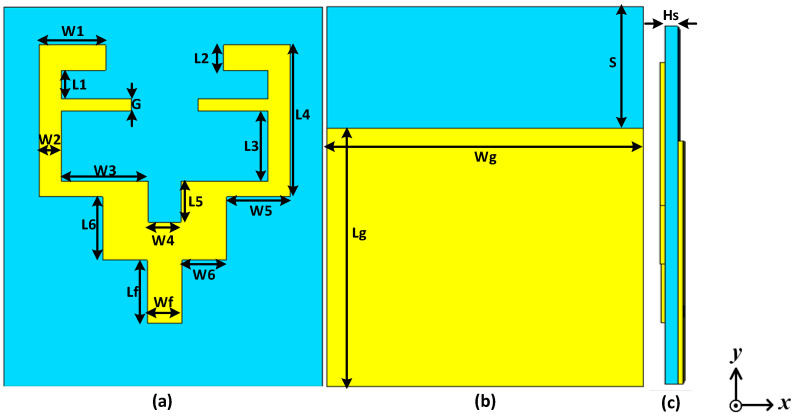
Structure of proposed triband antenna: (**a**) front view, (**b**) back view, and (**c**) side view.

**Figure 2:**
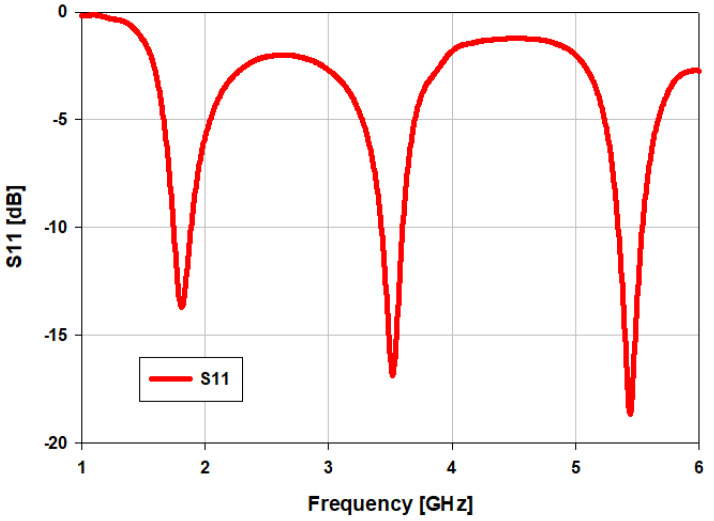
Reflection coefficient S_11_ of the proposed triband antenna.

Figure 1 illustrates the geometric configuration of the proposed triband flexible single element antenna. The planar patch is simple with two F-shaped resonators relative to a resonator at the lower and middle frequency bands while the middle-truncated patch helps operations at 5.4 GHz. Ground plane dimensions are reported as Lg × Wg = 50 mm × 40.8 mm; the dielectric material used above the rectangular ground plane is FR-4 possessing a height of Hs = 1.6 mm and a relative permittivity of *ε_r_* = 4.3. Generally, the overall dimensions of the designed antenna are 60 × 50 × 1.6 mm^3^. The antenna is fed by using a 50-ohm coaxial probe. The feed point is 2 mm above from the center of the feed patch. Figure 2 shows the simulated S_11_ of the triband antenna operating at 1.8 GHz, 3.5 GHz, and 5.4 GHz. The dimensions of the antenna are listed in Table 1. CST Studio Suite was used to simulate and analyze the performance of the antenna under bending conditions. CST Studio Suite is a powerful multilayer 3D full-wave electromagnetic solver that uses method of moments (MoM) technique to accurately solve Maxwell’s equations. The simulations include the thickness of the conductor.

### 2.2. Design Methodology

**Figure 3:**
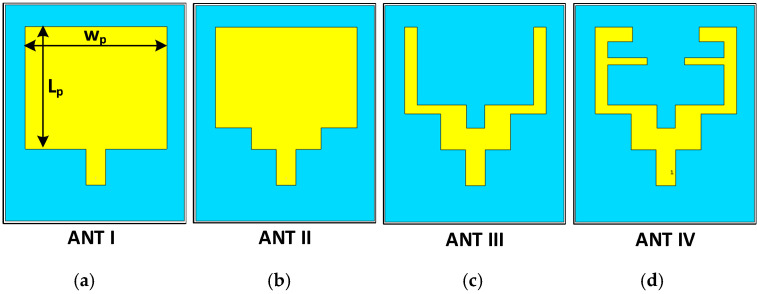
Design steps of the proposed triband antenna: (**a**) ANT I (patch only), (**b**) ANT II (Truncated patch), (**c**) ANT III (U-shaped patch), and (**d**) ANT IV (proposed antenna).

**Figure 4:**
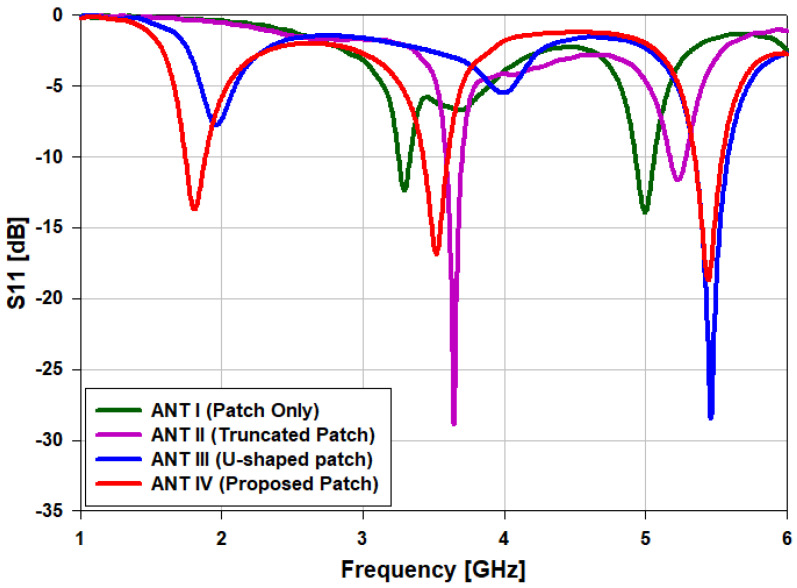
Simulated *S*_11_ for the corresponding design steps.

As illustrated in Figure 3, the proposed planar patch antenna for mobile applications is designed in four steps. First, in order to create a resonance band, a rectangular planar patch is designed to possess a width of Wp = 39.5 mm and a length of Lp = 41.5 mm centered at the middle of the FR-4 substrate. After that, a feed extension is designed to possess a length of Lf = 10 mm and a width of Wf = 5 mm starting from the center of lower edge of the rectangular planar patch in ANT I, as shown in Figure 3a. In this case, the MPA operated at 3.3 and 5 GHz. Then, in the next step, both bottom sides of the patch are truncated, and the patch width is kept unchanged in the case of ANT II, as presented in Figure 3b. A partial ground plane is created in each step of the design procedure, posessing a length of lg = 40.8 mm and width of wg = 50 mm. In the third step, a U-shaped patch is introduced, which is obtained from ANT II. Then, a small size rectangular slot is created in the middle portion of the planar patch, as shown in Figure 3c. In the final step, two F-shaped resonators are utilized, and the proposed planar patches are introduced (ANT IV), and each resonator has a length of L4 = 21.5 mm, as depicted in Figure 3d. The comparisons of S11 obtained from ANT I, II, III, and IV are illustrated in Figure 4.

The antenna design consists of a 50-ohm uniform coaxial probe, a ground plane, and a radiating patch. Based on the transmission line model, the dimensions of a rectangular patch antenna can be calculated with Equations (1)–(4) [26]. The width of the patch (*Wp*) can be calculated by using the following expression:

(1) $$Wp=\frac{\lambda_{o}}{2\sqrt{0.5\left(\varepsilon_{r}+1\right)}}$$

where *λ_o_* is the free space wavelength, and *ε_r_* is the substrate’s relative permittivity. The patch length *Lp* can be found by utilizing the following:

(2) $$Lp=\frac{c_{o}}{2f_{o}\sqrt{\varepsilon_{eff}}}-2\Delta L_{p}$$

where the light speed is *c_o_*, the extra length due to fringing effect is Δ*L_p_*, and *ε_eff_* is the effective dielectric constant. The *ε_eff_* can be found by using the following:

(3) $$\varepsilon_{eff}=\frac{\varepsilon_{r}+1}{2}+\frac{\varepsilon_{r}-1}{2}\left(1/\sqrt{1+12\frac{Hs}{W_{p}}}\right)$$

where *h* is the thickness of the substrate. The change in length resulting from fringing fields can be calculated by using the following.

(4) $$\Delta Lp=0.421h\left(\frac{\varepsilon_{eff}+0.3}{\varepsilon_{eff}-0.3}\right)\left(\frac{W_{p}/Hs+0.264}{W_{p}/Hs+0.813}\right)$$

The initial dimensions of the patch design at 1.8 GHz, 3.5 GHz, and 5.4 GHz using the above expressions for implementation on an FR-4 substrate of *εr* = 4.3 and *Hs* = 1.6 mm are *Lp* = 156 41.5 mm and *Wp* = 39.5 mm.

**Figure 5:**
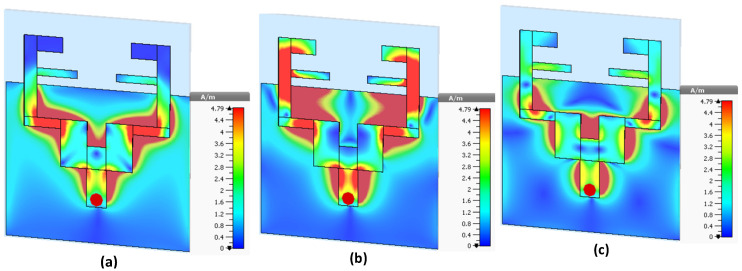
Surface current distribution (**a**) at 1.8 GHz, (**b**) at 3.5 GHz, and (**c**) at 5.4 GHz.

The current distributions at different frequencies can be observed in Figure 5. In order to better comprehend current density paths, the antenna has been separated into several regions. In Figure 5, the current has very low flow and magnitude in many regions, which means that the patch radiator is not excited at this frequency as expected. However, when the antenna operates at the desired frequency, the current flows have the largest magnitude. The current density of the triband antenna is shown in Figure 5. At the lower band (1.8 GHz), the current mostly flows around the T-shaped patch of the antenna, while surface current circulates in F-shaped resonators at 3.5 GHz, and some amount of current flows through the partial ground plane at 5.4 GHz.

### 2.3. Equivalent Circuit Model

**Figure 6:**
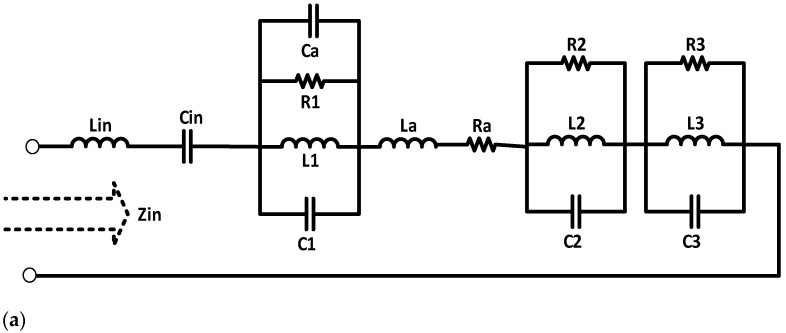

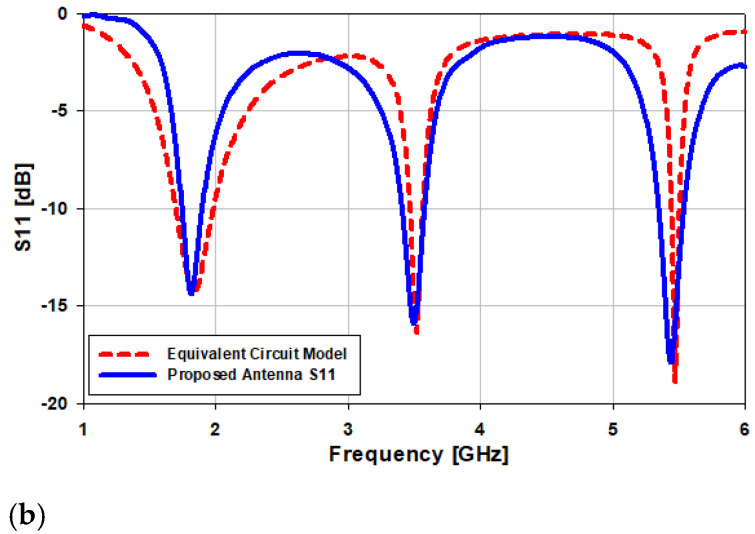
(**a**) Proposed equivalent circuit model for the triband antenna; (**b**) S_11_ obtained from the circuit model.

A circuit model for the triband MPA fed by a coaxial probe technique is designed using advanced design system (ADS) software. The main purpose of the equivalent circuit model is to validate the scattering parameters of the antenna as well as to prove that our proposed design is theoretically sound. The circuit model consists of five inductors, five capacitors, four resistors, and three resistor–inductor–capacitor (RLC) circuits connected in series with each other, as shown in Figure 6a. By varying the values of the resistors, the return loss of the circuit model can be varied, while the S_11_ of the MPA can be tuned by changing the values of the capacitors and inductors. The return loss of the circuit model is illustrated in Figure 6b. It covers bandwidths from 1.79 GHz to 2.01 GHz (220 MHz bandwidth) at 1.8 GHz; 3.36 GHz to 3.72 GHz (360 MHz) at 3.5 GHz; and 5.28 GHz to 5.52 GHz (240 MHz) at 5.4 GHz. The lumped element component values of the equivalent circuit model have been tabulated in Table 2.

## 3. Results and Discussion

### Fabrication and Measurements

**Figure 7:**
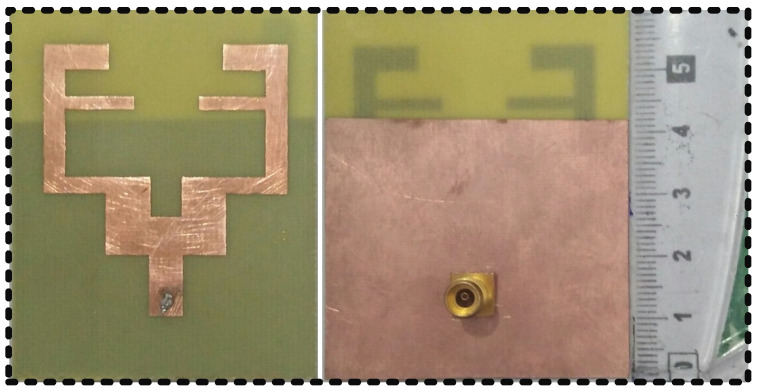
Front and back view of the fabricated prototype of the antenna.

**Figure 8:**
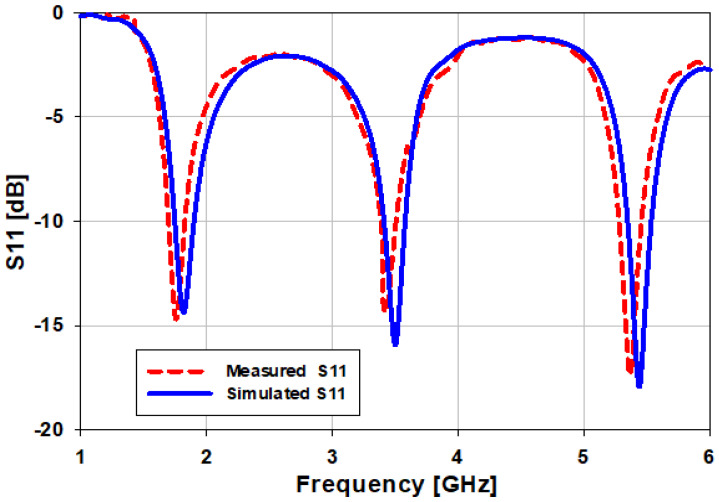
Simulated and measured return losses of the proposed antenna at three operating frequencies.

The proposed triband MPA was fabricated by using a computer numerical control (CNC) machine that specialized in PCB board manufacturing utilizing FR-4 substrate material (t = 1.6 mm). Figure 7 shows a fabricated antenna with a 50-ohm feed probe. The S_11_ performance of the triband MPA shows resonance frequencies at 1.8, 3.5, and 5.4 GHz with very good agreement with simulated ones, as in Figure 8. In the case of the simulation results, the S11 of the designed antenna covers the bandwidth from 1.74 GHz to 1.88 GHz (140 MHz or 7.7%); 3.42 GHz to 3.6 GHz (180 MHz or 5.14%); and 5.34 GHz to 5.54 GHz (200 MHz or 3.7%) at 1.8 GHz, 3.5 GHz, and 5.4 GHz, respectively, while in the case of measurement results, the antenna covers bandwidths 1.73–1.86 GHz (130 MHz or 7.22%) at 1.8 GHz; 3.4–3.54 GHz (140 MHz or 4%) at 3.5 GHz; and 5.2–5.45 GHz (250 MHz or 4.6%). 

**Figure 9:**
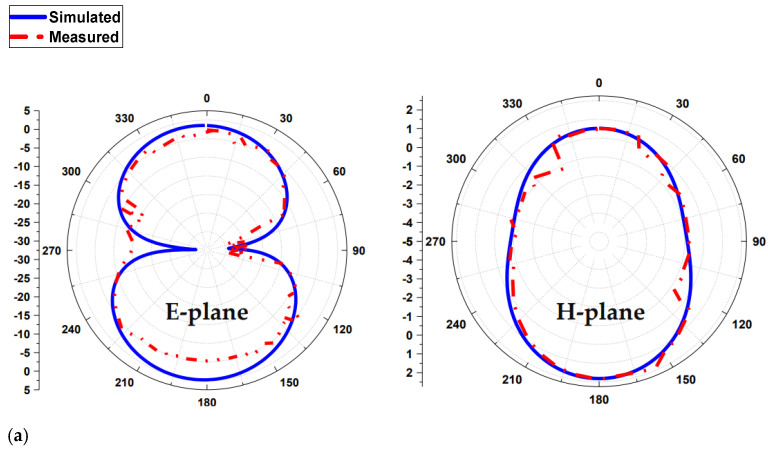

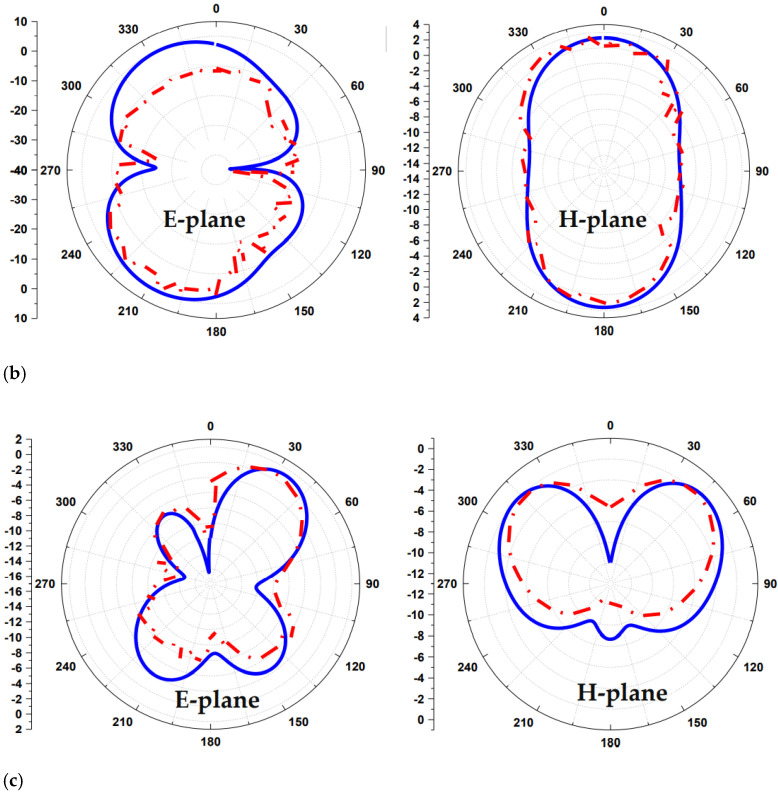
2D radiation pattern (**a**) at 1.8 GHz, (**b**) at 3.5 GHz, and (**c**) at 5.4 GHz.

The power transmitted in the direction of peak radiation relative to that of an isotropic source is referred to as antenna gain. The two-dimensional radiation pattern of the triband MPA is shown in Figure 9. The antenna has an elliptical shaped radiation pattern at 1.8 and 3.5 GHz, while a broadside directional pattern is obtained at a frequency band of 5.4 GHz. This antenna has been measured in an anechoic chamber by the Agilent N5227A vector network analyzer. The anechoic chamber is a reflection-free room and prevents surrounding waves from affecting ongoing measurements. This combination means that a person or detector perceives only direct sounds (no reverberant sounds), imitating the experience of being within an indefinitely large room. Anechoic chambers are structures that simulate testing in free space and are used to simulate and measure results including gains, S-parameters, and normalized antenna patterns.

Radiated efficiency compares the power delivered to the antenna terminals to the power radiated through the antenna as an electro-magnetic wave. If an antenna could be designed to be a perfect electrical component, it could convert all of the power provided to its terminals into radiating electromagnetic energy that could travel into space. This is only theoretically conceivable; thus, so some of the power sent to the antenna terminals is always lost in practice. For example, power losses are caused by a mismatch between the antenna element and the feeding network. Furthermore, the antenna material itself loses energy and generates undesired heat by its very nature. All of these losses add up to a situation where the antenna’s actual radiated efficiency is always less than 100% (equals 0 dB). By providing some power to the antenna feed pads and measuring the strength of the radiated electromagnetic field in the surrounding environment, antenna efficiency is measured in an anechoic chamber. In general, a good antenna transmits 50–60% of the energy provided to it (−3 to −2.2 dB).

**Figure 10:**
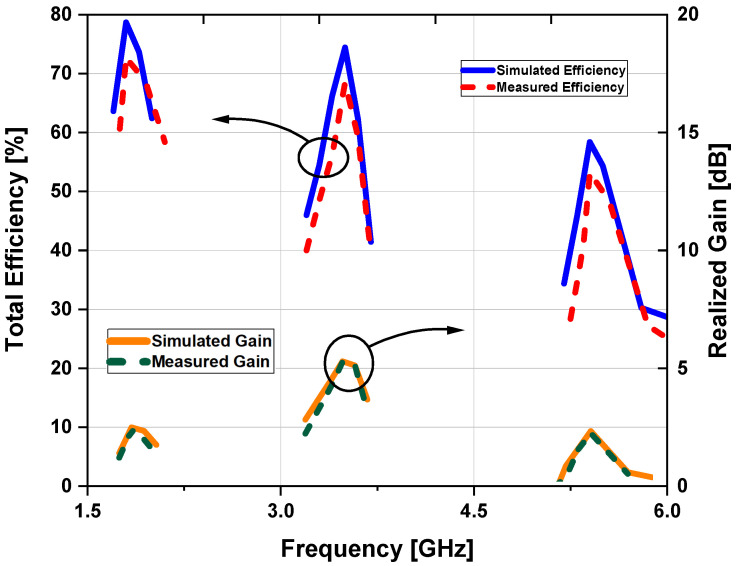
Simulated and measured gain and efficiency graph.

The simulated peak gain reported as 2.34, 5.2, and 1.42 dB at 1.8, 3.5, and 5.4 GHz frequency bands, respectively, while the measured realized antenna gain is calculated as 2.22 dB at 1.8 GHz; 5.18 dB at 3.5 GHz; and 1.38 dB at 5.4 GHz. Realized gains and efficiency vs. frequency are presented in Figure 10. In an anechoic chamber, antenna efficiency is determined by applying power to the antenna feed pads and measuring the intensity of the emitted electromagnetic field in the surrounding region. An efficiency of 73% at 1.8 GHz; 68% at 3.5 GHz; and 59% at 5.4 GHz is obtained experimentally.

## 4. Comparison with State-of-the-Art Antennas

The proposed antenna is compared with other reported multiband antennas in Table 3. The proposed antenna has a relatively small fractional bandwidth compared to the other antennas cited in Table 3. However, it offers higher gain and radiation efficiency performance. The main advantage of the proposed design is that it has higher gain, large bandwidth, compact size, simple design, and ease of implementation in practice. In addition to that, the low-cost material called FR-4 is used as a substrate, which means that the proposed antenna will be less costly but result in non-flexibility, as FR-4 is a duroid substrate with non-flexible materials.

## 5. Conclusions

A triband planar patch antenna with two F-shaped patches is designed, fabricated, and measured to operate at three bands, i.e., 1.8 GHz, 3.5 GHz, and 5.4 GHz. The proposed MPA is printed on a low-cost FR-4 substrate with a standard thickness of 1.6 mm. An equivalent circuit model is also designed to validate the reflection coefficient of the proposed antenna with the S_11_ obtained from the circuit model. A prototype of the presented triband MPA is built, and its characteristics are measured. Excellent agreements between the simulated and measured return loss results have been obtained. The antenna has an elliptically shaped radiation pattern at 1.8 GHz and 3.5 GHz, while a broadside directional pattern is obtained at the 5.4 GHz frequency band. Simulated peak-realized gains of 2.34, 5.2, and 1.42 dB are obtained at 1.8, 3.5, and 5.4 GHz respectively, while experimental peak realized gains of 2.22, 5.18, and 1.38 dB are obtained at the same frequencies. An efficiency of 73% at 1.8 GHz; 68% at 3.5 GHz; and 59% at 5.4 GHz is obtained experimentally. The results indicate that the proposed planar patch antenna can be utilized for wireless sensors in mobile applications. The antenna resonant frequency is suitable for distributed control system (DCS) applications at 1800 MHz, WiMAX applications at 3.5 GHz, and Wireless LAN applications at 5.4 GHz. The proposed patch antenna is a perfect candidate for wireless sensors in the applications of mobile phones.

## Figures and Tables

**Table 1:** Parameters of the proposed triband antenna.

Parameters	Value (mm)	Parameters	Value (mm)
Lg	40.8	Wg	50
L1	4.45	W1	10.5
L2	4.0	W2	3.5
L3	11.05	W3	13.70
L4	21.50	W4	5.10
L5	6.50	W5	10.0
L6	10.0	W6	7.0
Lf	10.0	Wf	5.0
G	2.0	S	19.2

**Table 2:** Lumped element component values of the equivalent circuit model.

Capacitor	Value (pF)	Inductor	Value (nH)	Resistor	Value (Ohm)
Cin	2.3	Lin	1	R1	50
C1	3.0	L1	1.3	R2	47
C2	20	L2	100	R3	45
C3	35	L3	24	Ra	2
Ca	2	La	1	Zin	50

**Table 3:** Comparison of the proposed antenna to other multiband antennas.

Ref. No.	Size (mm^3^)	Operating Frequency (GHz)	Bandwidth (MHz)	Peak Gain (dB)	Substrate Material	Proposed Technique
[27]	40 × 40 × 1.52	2.6, 6, 8.5	50, 22.8, 30	6.2, 4.52, 6.9	FR-4	Microstrip Patch
[31]	80 × 78.93 × 1.7	1.429, 1.839	NA	2.9, 4.3	FR-4	U-shaped patch
[33]	60 × 55 × 1.59	4.3, 5.0, 6.1, 7.4, 8.9, 9.2	68.6, 126.7, 132, 124.3, 191.2, 530.6	1.08, 3.23, 3.36, 2.77, 3.07, 4.87	FR-4	Square shaped microstrip patch
[34]	70 × 70 × 1.58	1.75, 3.65, 5.55, 6.6	170, 60, 140, 120	7.2, 11.2, 11.3, 7	FR-4	Sierpeinsiki-shaped patch
[35]	70 × 60 × 1.6	1, 1.2, 0.7	50, 60, 60	2.313, 2.396, 2.478	FR-4	Microstrip patch
[36]	94 × 78 × 3.18	2.53, 3.86, 6.45, 6.93	50.70, 410, 1250	8.18, 7.97, 10.56, 22, 5	Rogors RT5880	Microstrip Patch
[37]	50 × 50 × 1.5	1.2, 2.4, 5.6	12.76, 52.979, 52.979	NA	FR-4	Patch with defected ground
[This work]	60 × 50 × 1.6	Sim: 1.8, 3.5, 5.4 Meas: 1.7, 3.39, 5.38	Sim: 140, 180, 200 Meas: 140.2, 180.1, 200.2	Sim: 2.34, 5.2, 1.42 Meas: 2.22, 5.18, 1.38	FR-4	F-shaped Planar patch

## Data Availability

All data are included within the manuscript.
